# The Challenges of Analysing Highly Diverse Picobirnavirus Sequence Data

**DOI:** 10.3390/v10120685

**Published:** 2018-12-03

**Authors:** Matthew A. Knox, Kristene R. Gedye, David T. S. Hayman

**Affiliations:** Molecular Epidemiology and Public Health Laboratory (^m^EpiLab), Hopkirk Research Institute, Massey University, Private Bag 11-222, Palmerston North 4442, New Zealand; K.Gedye@massey.ac.nz (K.R.G.); D.T.S.Hayman@massey.ac.nz (D.T.S.H.)

**Keywords:** phylogenetics, polymerase gene, protein structure homology-modelling, *RdRp* gene, viral dark matter

## Abstract

The reliable identification and classification of infectious diseases is critical for understanding their biology and controlling their impact. Recent advances in sequencing technology have allowed insight into the remarkable diversity of the virosphere, of which a large component remains undiscovered. For these emerging or undescribed viruses, the process of classifying unknown sequences is heavily reliant on existing nucleotide sequence information in public databases. However, due to the enormous diversity of viruses, and past focus on the most prevalent and impactful virus types, databases are often incomplete. Picobirnaviridae is a dsRNA virus family with broad host and geographic range, but with relatively little sequence information in public databases. The family contains one genus, *Picobirnavirus*, which may be associated with gastric illness in humans and animals. Little further information is available due in part to difficulties in identification. Here, we investigate diversity both within the genus *Picobirnavirus* and among other dsRNA virus types using a combined phylogenetic and functional (protein structure homology-modelling) approach. Our results show that diversity within picobirnavirus exceeds that seen between many other dsRNA genera. Furthermore, we find that commonly used practices employed to classify picobirnavirus, such as analysis of short fragments and trimming of sequences, can influence phylogenetic conclusions. The degree of phylogenetic and functional divergence among picobirnavirus sequences in our study suggests an enormous undiscovered diversity, which contributes to the undescribed “viral dark matter” component of metagenomic studies.

## 1. Introduction

Emerging infectious diseases, particularly those of zoonotic origin, represent a significant burden to current and future human health [1,2,3]. The reliable identification and classification of emerging or undescribed infectious diseases is critical for understanding their biology, mitigating their impact and controlling their spread. For viruses, this process is heavily reliant on nucleotide sequence information. However, characterising virus species in laboratories, e.g., by amplifying viral agents in cell culture, antigenic/serological cross-reactivity or nucleic acid hybridisation to known viral sequences, is often difficult and time consuming [4], thus hindering the generation of well categorised molecular data. Accordingly, the majority of the virosphere is currently undescribed [5], leaving large gaps in knowledge of viral diversity [6]. That most viruses remain undescribed is unsurprising, considering that they are the most abundant entities on earth [7]. Furthermore, the key element used for identification (nucleic acid sequences, NAS) is replicated in several fundamentally different forms (single-stranded RNA [ssRNA], [ssDNA], double-stranded RNA [dsRNA], and [dsDNA]) such that no universal phylogenetic marker is available across all viruses [8]. At the other end of the taxonomic spectrum, individual viral taxa can evolve rapidly, presenting a moving target for molecular characterisation of specific types [9]. 

The advent of high throughput next generation sequencing technology has led to new fields of research, including viral metagenomics, which allows amplification of nucleotide sequences from any virus present in a particular sample without the need for isolation and/or culture of individual virus taxa. However, tools for the classification of viral NAS are reliant on databases, which are known to be incomplete. Viral metagenomic analyses of environmental samples suggest that the field of virology has explored less than 1% of the extant viral diversity [10]. Furthermore, the enormous sequence variation within viral families presents difficulties matching novel unclassified sequences, even with close relatives. This un-matched viral sequence, known as “viral dark matter” [11] can comprise up to 90% of the NAS in metagenomic samples and is especially prevalent in poorly characterised, abundant, widespread and highly diverse groups of viruses such as Picobirnaviridae. 

*Picobirnavirus* is a relatively recently established double-stranded RNA (dsRNA) virus genus associated with acute watery diarrhoea and gastroenteritis in humans [12,13,14] and animals [15,16]. However, it has also been detected in asymptomatic individuals [17] and invertebrates [18] and, alternatively, recent evidence suggests that picobirnaviruses may in fact infect prokaryotic [19] or fungal [20] host cells. Picobirnavirus was first observed in 1988 [21,22] and later formalised by establishing a new family, Picobirnaviridae, containing a new genus *Picobirnavirus*, with *Human picobirnavirus* as the type species, and *Rabbit picobirnavirus* as a designated species [23]. The *Human picobirnavirus RNA-dependent RNA polymerase* (*RdRp*) gene has been sequenced [24], leading to the identification of two distinct genogroups (I and II). Efforts to characterise the genome of picobirnavirus are hampered by the continued inability to culture this virus. The first picobirnavirus *RdRp* gene resulted in the development of primer sets for the PCR amplification of a 205 (PicoB25/43) and 368 (PicoB23/24) base pair (bp) fragment of the *RdRp* gene for genogroup I and II respectively [24]. Using these primer sets, picobirnavirus NAS fragments have been detected in faecal samples of many mammalian species including wild and domestic animals, as well as in wastewater samples [25,26,27,28]. In fact, of the 1057 sequences matching the query “Organism=picobirnavirus” on National Center for Biotechnology Information (NCBI), 707 were 210 bp or less and 513 were derived from the four publications above alone, which all use the primer pair developed by Rosen, Fang, Glass and Monroe [24]. Thus, the bulk of picobirnavirus sequences on repositories consist of fragmentary information derived from a limited number of sources. Complete genome sequences [29,30] or the full length genomic segment of *RdRp* gene [31,32] are also available for a handful of representatives, 38 of which are analysed in this study. 

Molecular studies on picobirnaviruses have found little congruence with host taxonomy [33], highlighting the lack of knowledge around picobirnavirus disease dynamics. To further complicate matters, multiple, distinct picobirnavirus genotypes have been amplified from the same host individual [32], suggesting high levels of diversity within picobirnavirus. Indeed, several studies have commented on the extremely high degree of sequence and amino acid incongruence among sequences identified as picobirnavirus [13,34,35], which may be as low as 49% similar [25]. Because of this uncertainly, we sought to investigate the current state of knowledge for picobirnavirus diversity both within the family and among other dsRNA virus types using sequences from NCBI. The aim of this work is to highlight issues around picobirnaviruses and dsRNA taxonomy. To do this, we use amino acid sequence alignments and phylogenetic analyses, and predicted protein secondary folding structure model comparisons to determine viral relationships.

## 2. Methods

Picobirnavirus *RdRp* sequences were obtained from the NCBI protein database and are derived from two previous analyses [18,32]. Complete *RdRp* sequences from a further six dsRNA families (representing 22 genera) were also downloaded from NCBI for comparison with the picobirnavirus sequences and can be found in Supplementary Material Table S1. Our focus with the additional families was on two families closely related to Picobirnaviridae (Birnaviridae *n* = 22 and Partitiviridae *n* = 70) and our aim was to compare and contrast these sequences using a range of phylogenetic methods based on amino acid sequence variation as well as with protein structure homology modelling. Analyses were conducted on three datasets (DS): DS1, 38 complete picobirnavirus *RdRp* sequences; DS2, 38 partial picobirnavirus *RdRp* sequences derived from DS1 (205 bp fragment from Rosen, Fang, Glass and Monroe [24]) and DS3, a combined dataset containing complete *RdRp* sequences from picobirnavirus (*n* = 38) and randomly selected *RdRp* sequences from other dsRNA families (*n* = 160). 

Picobirnavirus amino acid sequences (DS1-3) were aligned using MAFFT version 7 employing the E-INS-i algorithm [36]. All ambiguously aligned regions were then removed using the trimAl program [37], employing the gappyout setting. An untrimmed version of the full-length picobirnavirus alignment (DS1 untrimmed) was retained for later comparison with its trimmed counterpart. For each sequence alignment, the best-fit model of amino acid substitution was determined using ProtTest 3.4 [38]. Phylogenetic trees were subsequently inferred using the maximum likelihood approach (ML) implemented in PhyML version 3.0 [39], employing Subtree Pruning and Regrafting (SPR) branch-swapping. Branch support was estimated using an approximate likelihood ratio test (aLRT) with the Shimodaira–Hasegawa-like procedure implemented in PhyML. Due to the extreme diversity of the sequences analysed in our dataset, we do not use outgroups. ML trees were constructed for each dataset and resulting tree topologies were compared directly using cophylogeny plots and the weight of the difference in the trees estimated using the procrustean approach to cophylogeny [40] using ‘ape’, ‘phytools’ and ‘paco’ packages in R.

Protein structure homology-modelling using SWISS-MODEL [41] was carried out using the web interface and built with ProMod3 Version 1.0.2 (http://swissmodel.expasy.org/). Target picobirnavirus amino acid *RdRp* sequences were uploaded and appropriate templates found [42], with the closest match for all picobirnavirus sequences being 5i61.2.A, a human picobirnavirus *RdRp*. This template, generated from the crystal structure [43], was then used to build protein structure homology models for all 38 picobirnavirus *RdRp* amino acid sequences and *RdRp* sequences representing a further 22 dsRNA virus genera, enabling a comparative analysis of protein structure. We compared the resulting QMEAN, Global Model Quality Estimate (GMQE) and sequence similarity for all sequences. QMEAN [44,45] is a composite scoring function based on different geometrical properties and accounts for both global (i.e., for the entire protein structure) and local (i.e., per residue) absolute quality estimates in a single output score. Higher QMEAN scores indicate better agreement between the model structure and experimental structures (templates) of similar size and scores of -4.0 or below indicate poor matches. GMQE is a model quality estimation and is based on combined properties of the target-template alignment and the template search method. The resulting GMQE score is expressed as a number between 0 and 1, with higher numbers indicating higher reliability and reflecting the expected accuracy of a model built with that alignment and template [42]. 

To investigate protein structure homology-modelling analyses further using other, potentially better-characterised dsRNA virus families, we repeated the approach on *RdRp* amino acid sequences in our dataset belonging to the Birnaviridae family. We used the template 2yib.1.A and compared the resulting QMEAN, GMQE and sequence similarity. Template matches were found using the same process as for picobirnavirus using SwissModel search strategies [42]. Unlike picobirnavirus, the resulting template matches were not the same for all sequences within a family/subfamily, though they were the same within genus. This probably reflects the greater number of templates available for viruses within Birnaviridae. For consistency with the Picobirnaviridae analysis, one template was chosen per family. A similar analysis was attempted for Partitiviridae, but no close template matches could be found using SwissModel (i.e., no QMEAN > −4 for any genus using any template). 

Finally, we investigated the effect of trimming strategies on phylogenetic and functional comparisons made for DS3. Alignments were generated as outlined above, though in this case we retained untrimmed alignments and alignments generated with both the gappyout and strict settings in TrimAl. Again, the best-fit models of amino acid substitution were determined and phylogenetic trees generated using the same processes as described above. We explore the effects of trimming on resulting protein structure homology-modelling by comparing amino acid sequences to the picobirnavirus template 5i61.2.A.

## 3. Results

**Figure 1:**
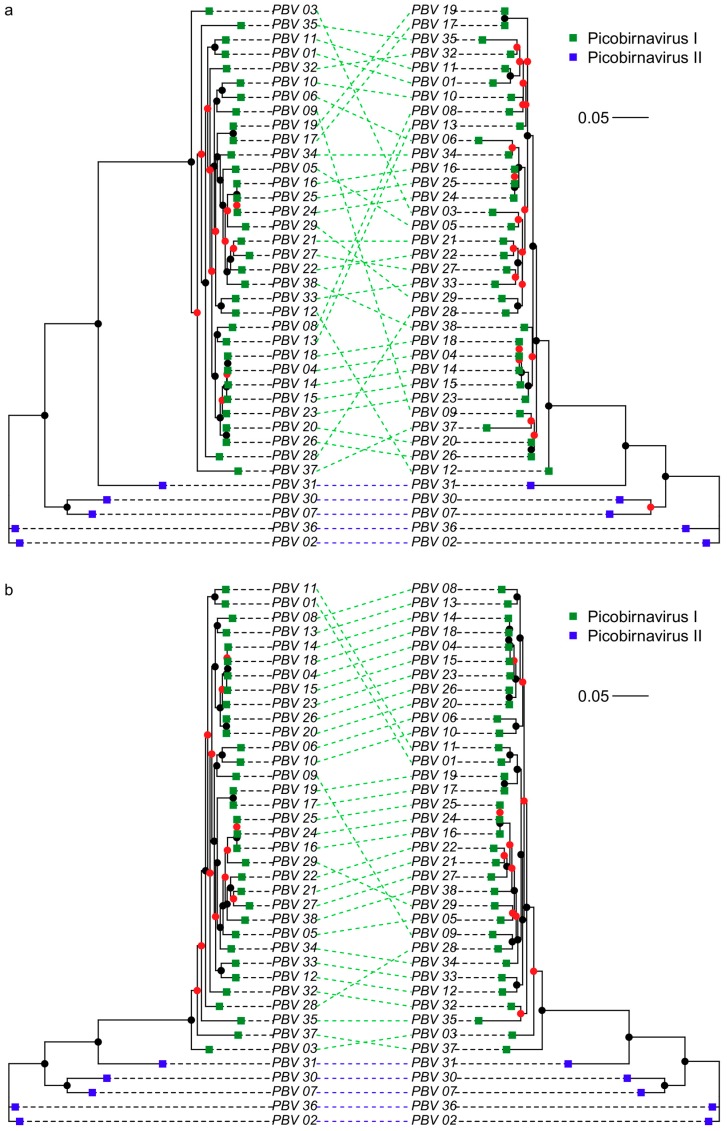
Cophylogeny ML trees of (**a**) trimmed complete v trimmed partial *RdRp* picobirnavirus amino acid sequences and (**b**) trimmed complete v untrimmed complete *RdRp* picobirnavirus amino acid sequences. Scale bar corresponds to 0.05 substitutions per amino acid. Branch support values are presented as coloured nodes (black > 0.8, red < 0.8).

**Figure 2:**
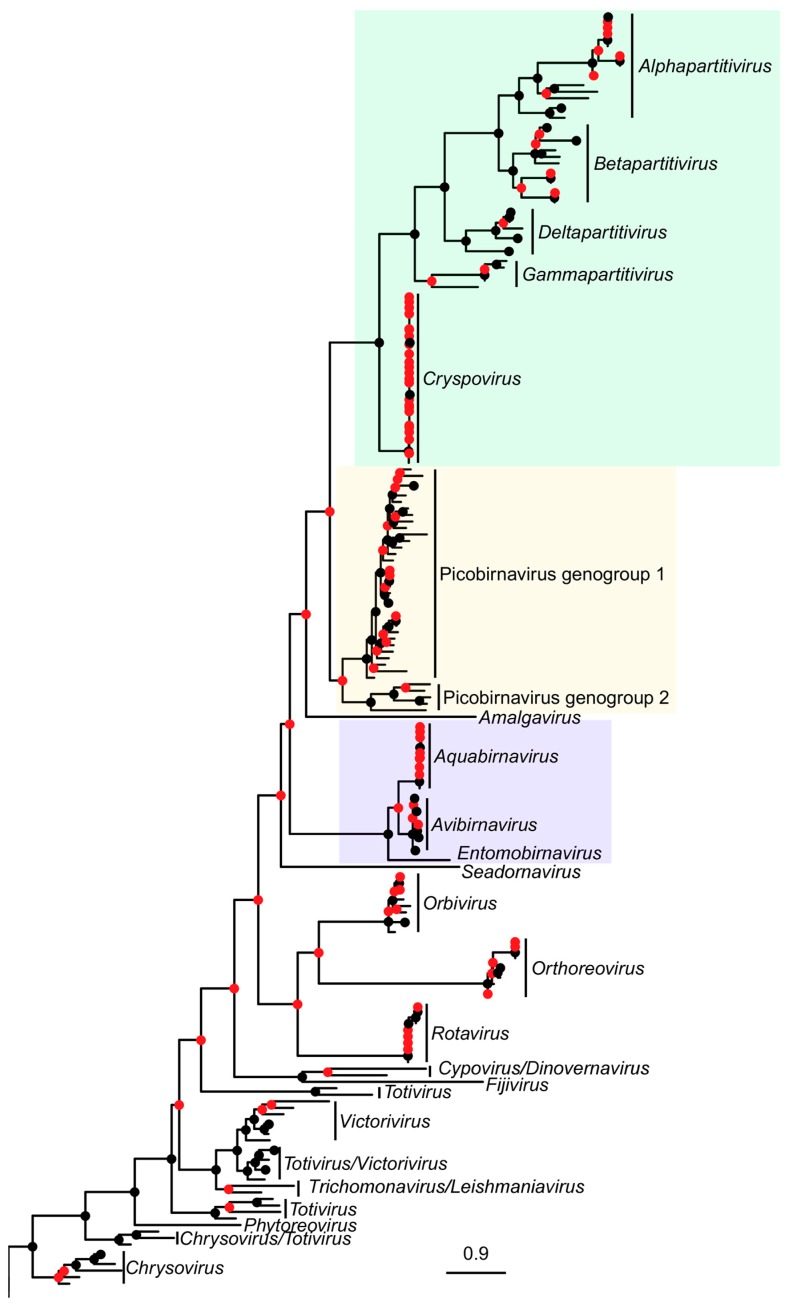
Maximum likelihood phylogenetic tree of *RdRp* amino acid sequences from picobirnavirus and representatives from other dsRNA virus families. Scale bar corresponds to 0.9 substitutions per amino acid. Putative genogroups I and II are shown in the Picobirnaviridae (gold shaded) portion of the tree. Related families Partitiviridae (green) and Birnaviridae (blue) are also shaded. Branch support values are presented as coloured nodes (black > 0.8, red < 0.8).

The following parameters were found using PhyML and used in the construction of maximum likelihood trees: RtREV+I+G+F, I = 0.046, G = 1.016 (DS1-trimmed), RtREV+I+G+F, I = 0.044, G = 1.013 (DS1-untrimmed), LG+I+G, I = 0.069, G = 1.075 (DS2) and LG+I+G, I = 0.017, G = 1.176 (DS3 trimmed (gappyout)). TrimAl processing in DS1 reduced sequence length by between 1.1% and 8.7% (mean reduction 3.7% in Supplementary Material Table S2) and caused a rearrangement of phylogeny (Figure 1a). Our cophylogeny plots also demonstrated altered phylogenetic relationships among picobirnavirus sequences when comparing partial and complete *RdRp* sequences (Figure 1b). The phylogenetic relationships within picobirnavirus and relative to other dsRNA groups are examined in Figure 2. Many of the branches for distantly related dsRNA virus families such as Totiviridae, etc., are poorly supported and resolved, reflecting the massive diversity of dsRNA *RdRp* genes (Supplementary Material Figure S2). Picobirnavirus can clearly be split into two distinct clades (picobirnavirus 1 and 2 in Figure 2), representing the putative genogroups I and II. The sequences from other dsRNA virus families are presented here to give an estimate of phylogenetic diversity within and between these groups and demonstrate that Picobirnaviridae appears to have high levels of within-genus sequence divergence, relative to many other dsRNA virus families, e.g., Birnaviridae, but similar to levels of divergence seen within genera in others, e.g., *Alphapartitivirus*. 

**Figure 3:**
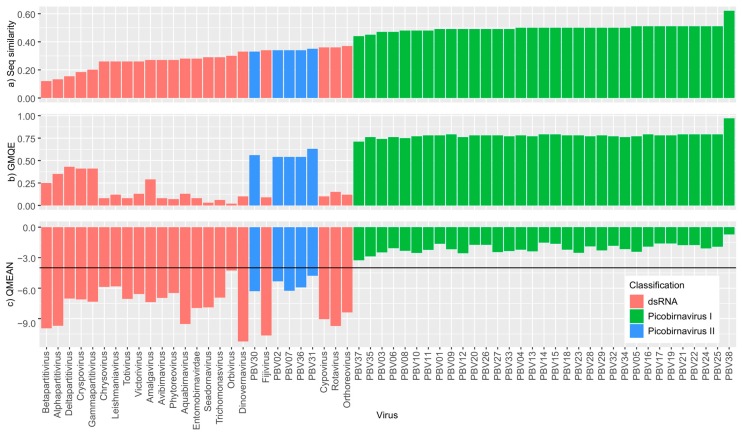
Swiss Model results (**a**) sequence similarity to template, (**b**) Global Model Quality Estimate (GMQE) and (**c**) QMEAN for all sequences. Higher QMEAN scores indicate a better match to the template and the horizontal line in the QMEAN graph at −4 indicates the model quality cut off point.

**Figure 4:**
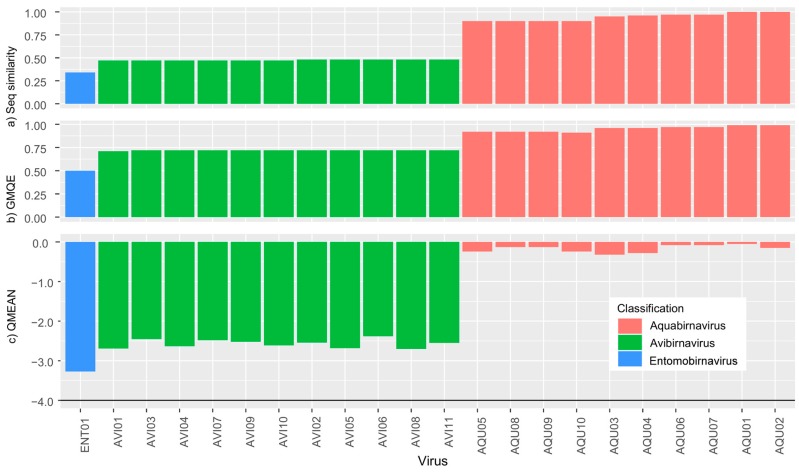
Swiss Model results for (**a**) sequence similarity, (**b**) Global Model Quality Estimate (GMQE) and (**c**) QMEAN for sequences from Birnaviridae. Higher QMEAN scores indicate a better match to the template and the horizontal line in the QMEAN graph at −4 indicates the model quality cut off point.

Our protein structure homology-modelling analyses revealed a distinct split in picobirnavirus, with genogroup I sequences achieving higher QMEAN scores than either genogroup II or other dsRNA sequences (Figure 3) indicating a closer match to the template. These findings are expected since the template used for the analyses was derived from a genogroup I picobirnavirus. However, the QMEAN values of picobirnavirus genogroup II sequences are similar to those of other dsRNA families (Figure 3c), i.e., <−4 (a very poor fit [44]). The GMQE scores for all picobirnavirus samples identified as genogroup I fell within 0.71 and 0.79, with the exception of PBV38 (GMQE = 0.97), which reflects its very close similarity to the template. Genogroup II sequences had lower scores, falling between 0.54 and 0.63, indicating lower reliability and model accuracy of these sequences. The remaining dsRNA virus family sequences all had very low GMQE scores (0.02–0.29). Our analysis of Birnaviridiae resulted in different patterns to those observed in picobirnavirus. The QMEAN values were all <3.3, indicating a close match to the template across the three genera within the family (*Aquabirnavirus*, *Avibirnavirus*, *Entomobirnavirus*) (Figure 4). Similar to Picobirnaviridae results, GMQE and sequence similarity scores were inversely correlated to QMEAN values. 

**Figure 5:**
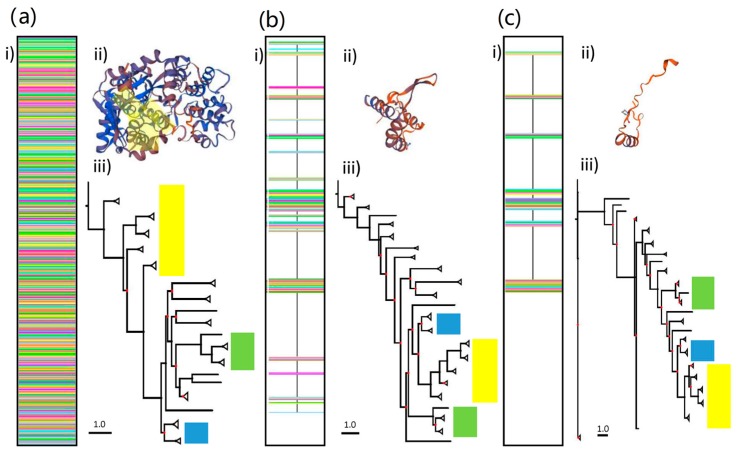
Alignment (i), protein structure homology-modelling analyses (ii) and maximum likelihood phylogenetic trees (iii) for (**a**) untrimmed, (**b**) trimmed-gappyout and (**c**) trimmed-strict amino acid sequences using YP_239361.1 (534 amino acid picobirnavirus genogroup 1 sequence). Scale bar corresponds to 1.0 substitutions per amino acid. Highlighted areas in a (ii) show protein structure regions retained in b and c. Highlighted regions in (iii) show the relative position of genera from Partitiviridae (yellow), Birnaviridae (green) and Picobirnaviridae (blue) families. Branch support values in trees are presented as coloured nodes (black > 0.8, red < 0.8).

Due to the extremely broad range of taxa involved in the analysis, the untrimmed DS3 alignment was 3,490 amino acids long and contained a very high proportion of gaps. Trimming using TrimAl overcame this issue, but reduced the alignment length to 116 and 69 amino acids using the gappyout and strict settings respectively. The retained regions were located in several small segments of amino acids located across the length of the complete *RdRp* gene (Figure 5a). Despite the extensive trimming, the retained regions maintained some level of compatibility with the template used previously (5i61.2.A) (Figure 5b). Maximum likelihood trees constructed using the following parameters: VT+I+G+F, I = 0.003, G = 2.983 (DS3-untrimmed), LG+I+G I = 0.017 G = 1.176 (DS3-trimmed gappyout), LG+I+G, I = 0.028 G = 1.141 (DS3-trimmed strict), reveal contrasting phylogenies. As was the case with the previous full *RdRp* gene analyses, many of the branches for distantly related dsRNA virus families in Figure 5 are poorly supported in partial and untrimmed trees (Supplementary Material Figure S3). We focus on the positions of Picobirnaviridae, Birnaviridae and Partitiviridae relative to each other and the remaining DS RNA virus groups and find large differences between trimmed and untrimmed phylogenies, as well as differences between gappyout and strict trimming methods (Figure 5c).

## 4. Discussion

In developing countries, diarrhoea is the most common cause of death in children under 5 years old, and this can be linked to a wide variety of pathogens. However, the etiologic agents of up to 40% diarrheic cases are unknown [46] despite extensive diagnostic analyses, suggesting a large undescribed component of disease burden. Metagenomic studies provide access to previously unsequenceable genetic information, but rely on existing taxonomic framework to characterise the sequence information that they generate. In order to gain a better understanding of the diversity and taxonomy of picobirnaviruses, a potential part of this undescribed component, we have analysed available sequence data using phylogenetic and comparative protein structure approaches. We wished to assess diversity within picobirnavirus relative to other dsRNA families and demonstrate that by analysing the data in different ways, we generate different conclusions from the same data.

Improved sequencing technologies have triggered a recent wave of biodiversity discovery for RNA viruses, revealing some of the hidden diversity and highlighting major gaps in phylogeny [6,18]. The results of our analyses provide further evidence of the high degree of genetic diversity within and among picobirnavirus genogroups I and II [13]. Our phylogenetic analyses show that the genetic distance between genogroup I and II sequences exceeds that of currently recognised genera within other dsRNA families. For example, average pairwise distances between *Avibirnavirus* and *Aquabirnavirus* sequences within the family Birnaviridae are 31.8% compared with 58.4% between picobirnavirus genogroups 1 and 2. The International Committee on Taxonomy of Viruses have stated that viruses that are identified solely from their genome information should be included in the taxonomic framework [47]. Based on analyses of genomic data in this and other studies, it appears that genogroups I and II qualify as distinct genera within Picobirnaviridae. Furthermore, the recent identification of other, highly divergent genogroups (III) within picobirnavirus [48,49] suggests that the diversity of picobirnavirus is even more under-represented in databases than currently thought. As more sequence information becomes available, Picobirnaviridae may be divided into distinct genera along these lines. Other, better-characterised dsRNA virus genera, e.g., *Avibirnavirus* and *Aquabirnavirus,* have distinct hosts (chicken and salmonids respectively). However, the lack of congruence between observed phylogenetic relationships and putative host as well as geography present difficulties establishing species in Picobirnaviridae [32]. Indeed, recent evidence suggests that picobirnavirus may not infect eukaryotic hosts at all, based on the presence of a classical bacterial sequence motif, the ribosomal binding site, previously only observed in viruses infecting prokaryotes [19]. If picobirnaviruses are prokaryotic viruses, then their diversity (i.e., genogroups) may reflect the phylogeny of bacteria found in various mammal hosts.

Despite picobirnavirus possessing a relatively short genome (~4 kb), much of the genetic information on public databases, i.e., available for classifying sequences, is based on a ~200 bp segment of the *RdRp* gene. As discussed above, levels of sequence divergence among *Picobirnavirus* sequences in databases are large, and for this reason we compared cophylogenies based on complete *RdRp* gene sequences and corresponding trimmed partial gene fragments. Our findings suggest that the short sequences commonly used for identification of *Picobirnavirus* are not always reflective of entire *RdRp* gene phylogeny and could therefore lead to incorrect conclusions. We acknowledge that branch support values for many of the internal nodes within genogroup I are low (<0.8, Figure 1, Supplementary Material Figure S1) meaning that some of the rearrangements occur because of poorly resolved tree structure. Nonetheless, some taxa (e.g., PBV03) belong to well supported clades and appear in different areas of the trees in Figure 1a. Therefore, taxonomic identifications, evolutionary relationships and viral source attribution based on short picobirnavirus sequences should be treated with some caution. Despite these cautions however, our protein structure homology-modelling analyses provide some support for the use of a short region in broad taxonomic classification. The region includes three of the six sections of amino acids retained following strict trimming (Supplementary Material Figure S4A) and retains the same functional structures (Supplementary Material Figure S4B), suggesting it is conserved and taxonomically informative. Interestingly, the short *RdRp* sequences amplified with the PicoB25/43 primer pair [24] are often unable to generate matches with reference sequences in databases. For example, of the 288 sequences generated by Symonds, Griffin and Breitbart [27], 28% did not closely match anything in NCBI databases. This could be caused by non-target amplification or amplification of multiple distinct picobirnavirus sequences. However, the unmatched fraction may also potentially reflect the incompleteness of current databases and represent novel picobirnavirus strains, similar to the viral dark matter encountered in metagenomic studies [46]. 

The high degree of sequence dissimilarity presents difficulties in alignment of sequences, even from within a single virus family. In such cases, trimming tools, such as TrimAl program [37], remove poorly aligned regions and allow more robust phylogenetic comparisons. However, the sequence reads used in BLAST or metagenomic database search approaches do not undergo trimming before analyses and may thus give inaccurate matches, especially in poorly resolved taxa. To investigate the potential impact of this, we compared phylogenies resulting from trimmed and untrimmed picobirnavirus sequences. Unsurprisingly, the majority of trimming occurred among genogroup II picobirnavirus sequences (Supplementary Material Table S1) as these were the most divergent and therefore contained a higher proportion of phylogenetically uninformative sequence information. Nonetheless, our findings suggest that the untrimmed genogroup I sequences have a different phylogenetic structure to trimmed, potentially leading to similar discrepancies to those explored above for complete vs. partial sequences. The degree of trimming is greatly increased when including representatives from a broader taxonomic range, as is the case in DS3. Here, we included amino acid sequences from seven DS RNA families and the resulting alignments were reduced by up to 80% of the original sequence length, and broken into several regions. The areas that were retained likely correspond to the most conserved *RdRp* regions across the range of dsRNA virus families we have analysed. The structure of the picobirnavirus *RdRp* is described in Collier, Lyytinen, Guo, Toh, Poranen and Tao [43] and comparison with information in Figure 1 in this paper show that ‘finger’ regions β3, β4, β5, α10 and ‘palm’ regions α11, α12 α16 of the core polymerase domain are retained in our trimmed sequences. The N- and C-terminal domains and flexible insertion loop structure are not present, nor is the ‘thumb’ subdomain. 

Like many other viral taxa, structural aspects of picobirnavirus virion (two genome segments, icosahedral non-enveloped capsid) are the primary criteria used to define its taxonomic status. NAS provide additional support for taxonomy but do not necessarily reflect functional aspects of an organism and to address this, we have analysed protein structure. The recent advances in protein structure homology-modelling allow insight into the functional properties of proteins derived from amino acid sequences [41]. Protein structure homology modelling can be used to explore the effect that differences in sequences have on the actual expressed structures of a gene. For instance, there may be structures, common to taxonomic groups, which could be useful in classification. Further, since protein structure homology modelling does not require alignments or trimming, it allows direct comparison of biologically meaningful structures. Our study did not seek to comment on the potential functions of the proteins that we have analysed and our findings should be treated with some caution as GMQE scores (a measure of model quality) were particularly low for the other dsRNA family sequences we analysed. Indeed, this would be inappropriate since only picobirnavirus genogroup I were of sufficient similarity to the template to allow such an analysis to be carried out. Instead, we sought to demonstrate that the diversity in *RdRp* sequences within picobirnavirus was comparable to that seen among genera in better characterised dsRNA virus groups. Our comparative protein structure analyses (QMEAN scores) suggest that the functional differences of genogroup II picobirnavirus are comparable to those seen among genera from other, distinct dsRNA viral families such as *Leishmaniavirus*, *Orbivirus*, *Phytoreovirus* and *Trichomonasvirus*. In comparison, the QMEAN scores for three genera within family Birnaviridae were all above −4 when compared with a different template, suggesting that diversity within these genera is low relative to picobirnavirus and providing further evidence that picobirnavirus genogroups may require formal taxonomic revision. 

Classification of NAS reads in metagenomic studies and other research using molecular information relies on comparison with reference sequences in databases such as NCBI. Our analyses highlight the caution required in classifying viruses through NAS fragment analyses and show how comparative protein structure homology-modelling can be used to explore functional aspects concurrent with phylogenetic approaches. Despite the incompleteness of reference databases and limitations with identification, picobirnavirus sequences appear increasingly frequently in metagenomic samples derived from a wide range of distinct environments, hosts and geographic ranges [48,50,51,52,53,54,55,56,57]. However, these data have yet to provide a solid understanding of the full breadth of picobirnavirus sequence diversity or pathogen–host relationships. Future research examining these secondary classification criteria may be used to define genera and resolve the question of what is a picobirnavirus?

## Supplementary Materials

The following are available online at http://www.mdpi.com/1999-4915/10/12/685/s1. Figure S1: (A) trimmed complete, (B) trimmed partial and (C) untrimmed complete trees used in Figure 1 cophylogeny showing branch support values. Figure S2: Maximum likelihood phylogenetic tree shown in Figure 2 with branch support values. Figure S3: (A) untrimmed, (B) gappyout trimmed and (C) strict trimmed trees used in Figure 5 showing branch support values. Figure S4: (A) Alignment of (i) untrimmed, (ii) trimmed strict and (iii) section of amino acid sequences amplified with primer set in Rosen, Fang, Glass and Monroe [23] (Picobirnavirus genogroup 1) for complete *RdRp* gene sequence YP_239361.1. (B) protein structure homology-modelling analyses for above sequences using the template 5i61.2.A. Table S1: Picobirnavirus sequence information, SwissModel results and TrimAl statistics. Table S2: Other DS RNA families used in DS3 analyses.
