# Supplementary material for: The Challenges of Analysing Highly Diverse Picobirnavirus Sequence Data
Source: Viruses. 2018 Dec 3;10(12):685. doi: 10.3390/v10120685 (PMC6316005; doi:10.3390/v10120685)
Supplement: Supplementary file 1 [file viruses-10-00685-s001.zip › SI/Figure S4.pptx]

## Slide 1
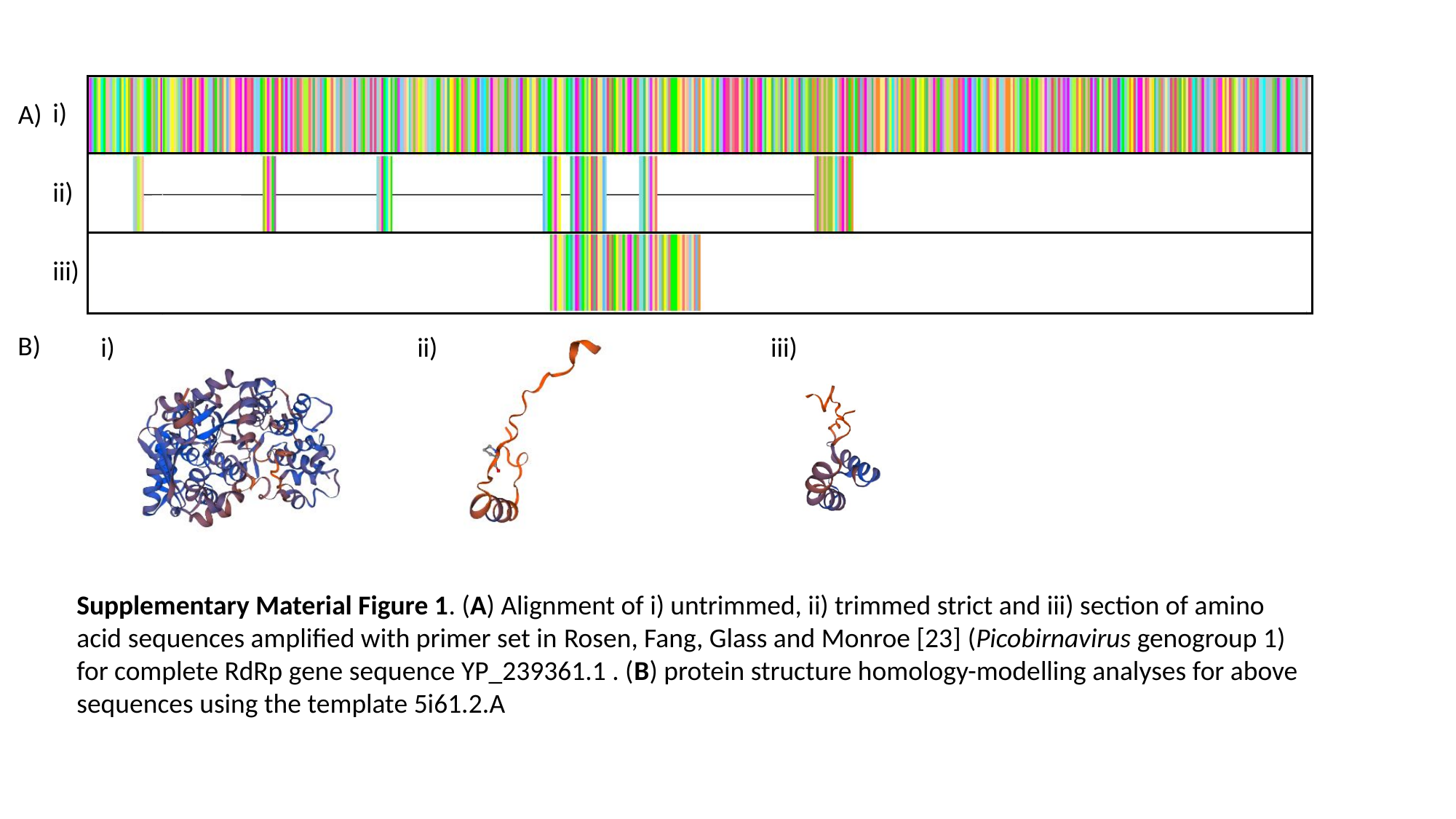

i)
ii)
iii)
A)
B)
i) ii) iii)
Supplementary Material Figure 1. (A) Alignment of i) untrimmed, ii) trimmed strict and iii) section of amino acid sequences amplified with primer set in Rosen, Fang, Glass and Monroe [23] (Picobirnavirus genogroup 1) for complete RdRp gene sequence YP_239361.1 . (B) protein structure homology-modelling analyses for above sequences using the template 5i61.2.A
