# Supplementary figures and images for: The Challenges of Analysing Highly Diverse Picobirnavirus Sequence Data
Source: Viruses. 2018 Dec 3;10(12):685. doi: 10.3390/v10120685 (PMC6316005; doi:10.3390/v10120685)

0.1

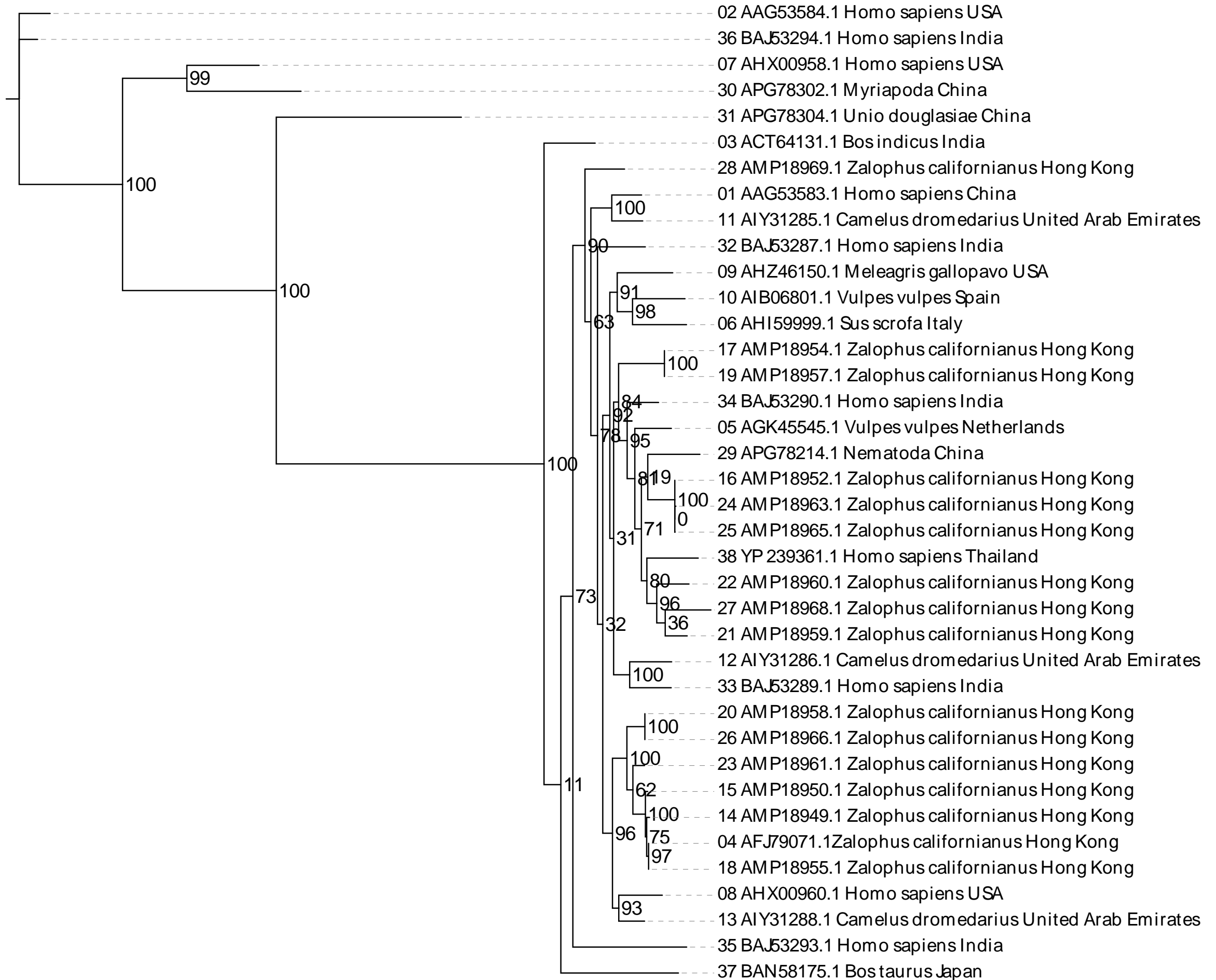

Supplement: Supplementary file 1 [file viruses-10-00685-s001.zip › SI/Figure S1_A.pdf]

0.1

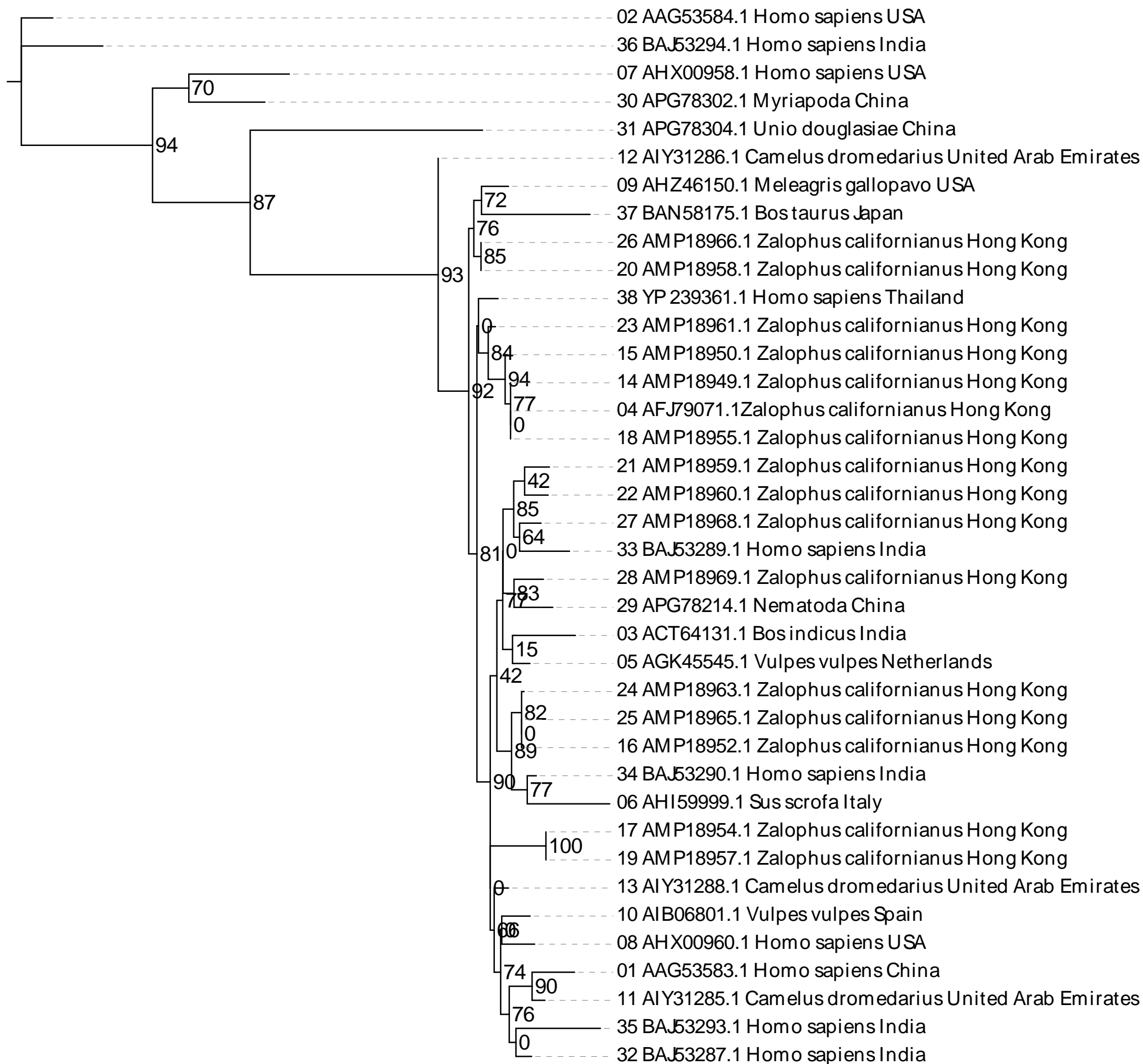

Supplement: Supplementary file 1 [file viruses-10-00685-s001.zip › SI/Figure S1_B.pdf]

0.1

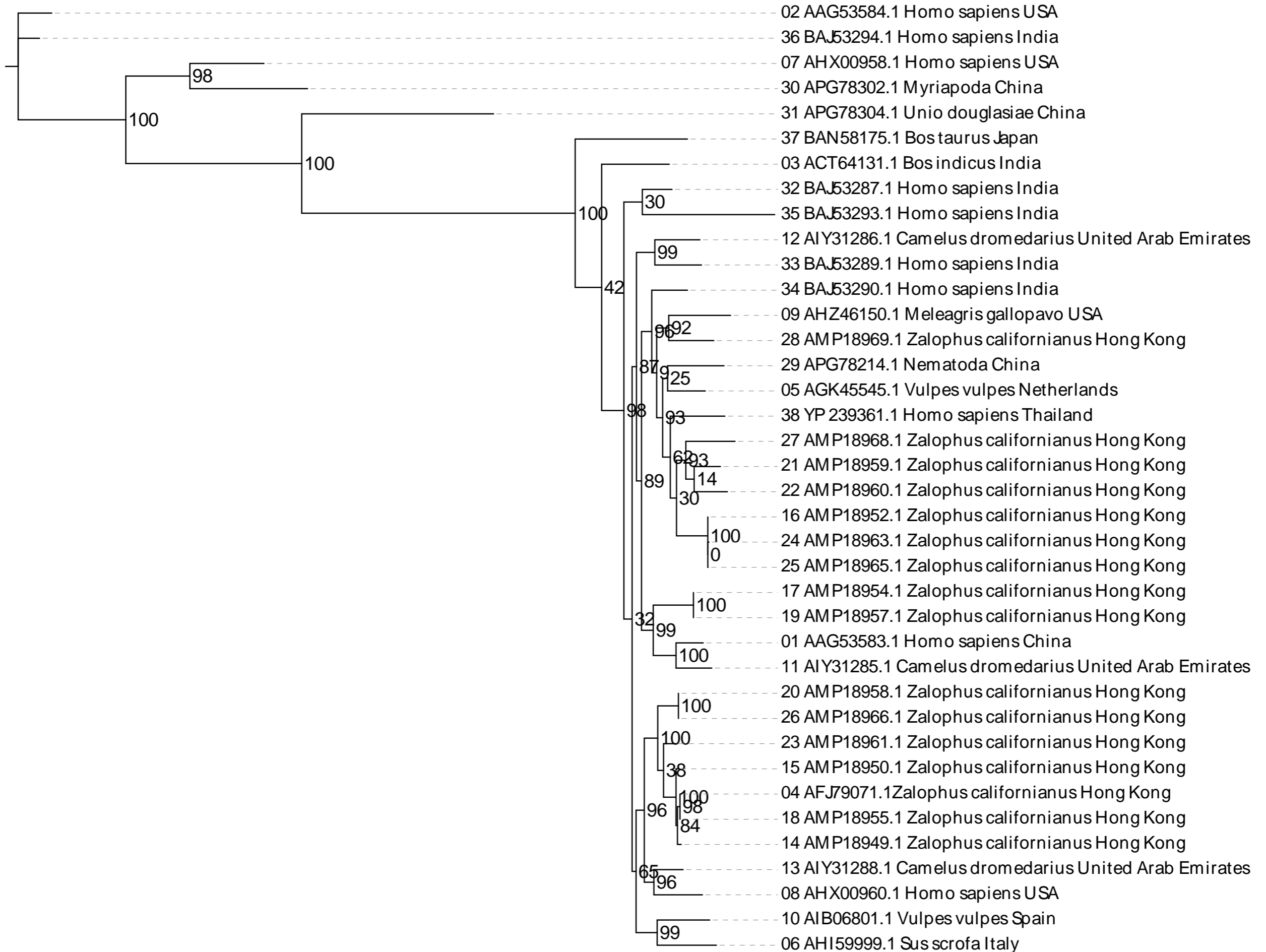

Supplement: Supplementary file 1 [file viruses-10-00685-s001.zip › SI/Figure S1_C.pdf]

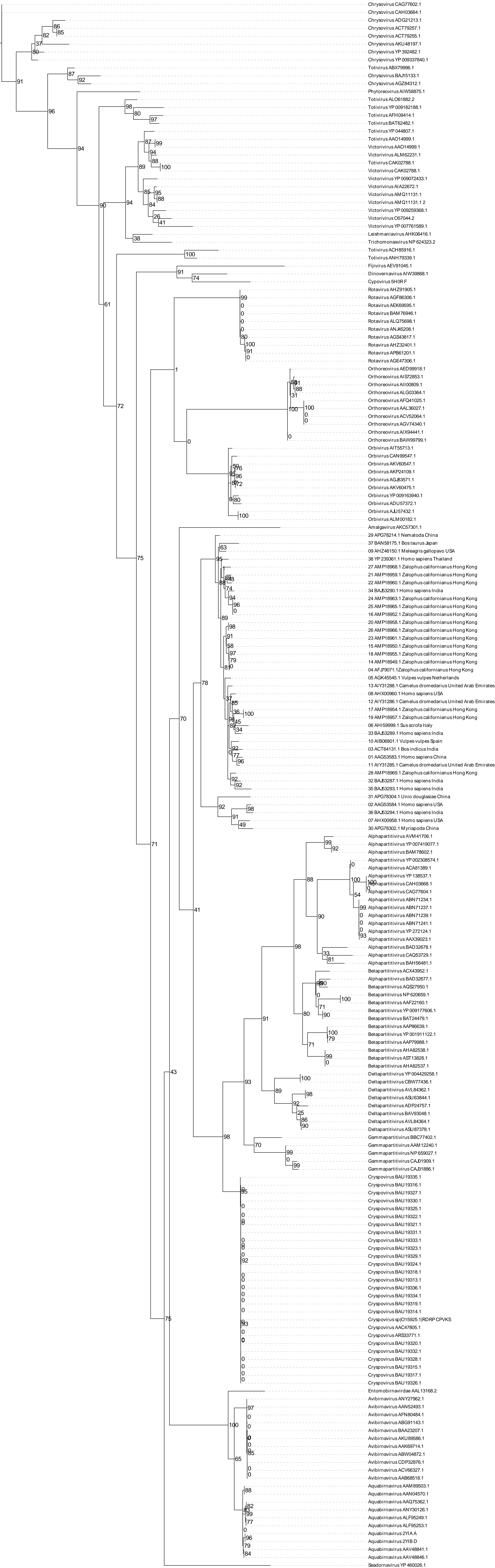

Supplement: Supplementary file 1 [file viruses-10-00685-s001.zip › SI/Figure S2.pdf]

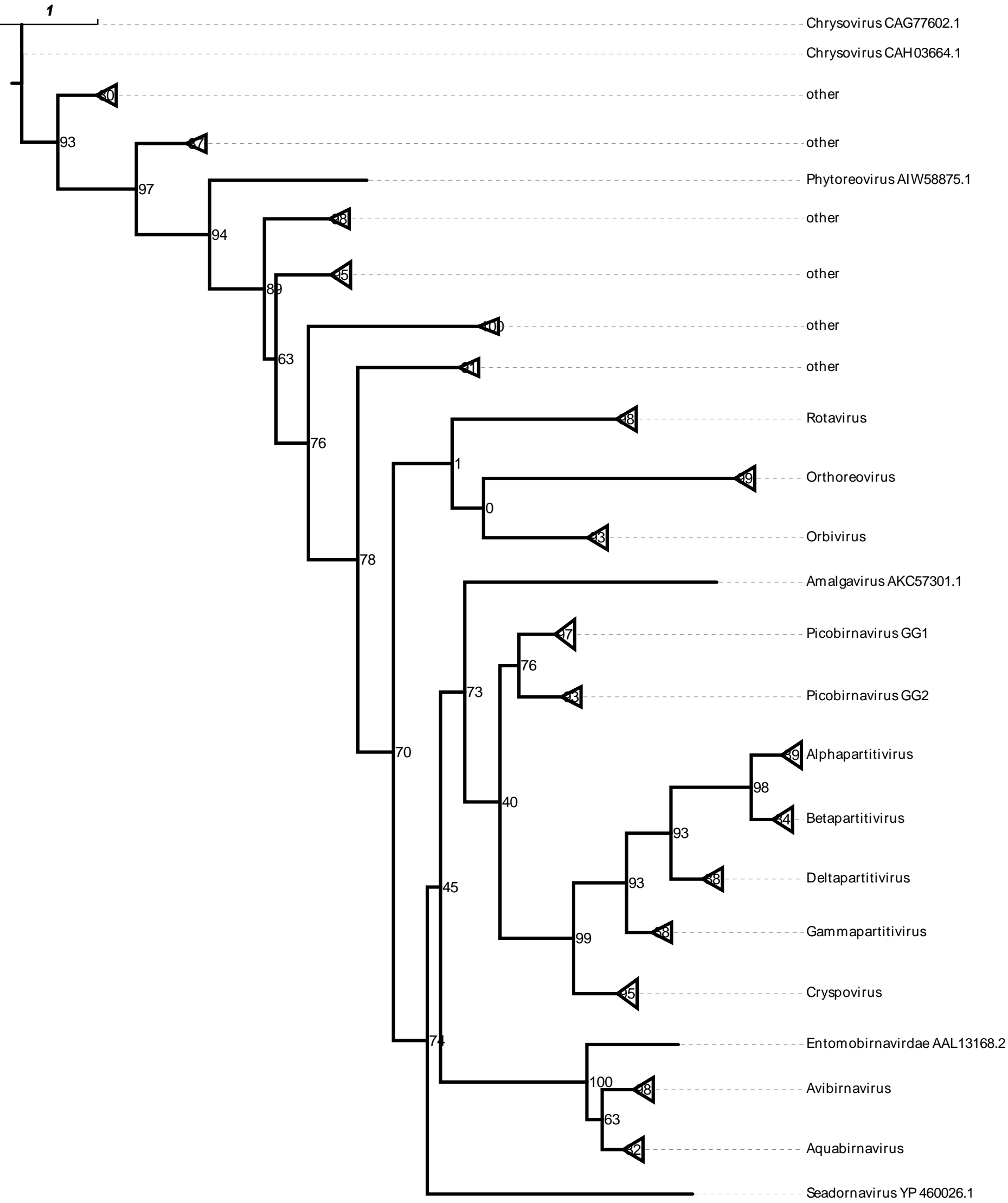

Supplement: Supplementary file 1 [file viruses-10-00685-s001.zip › SI/Figure S3_B.pdf]

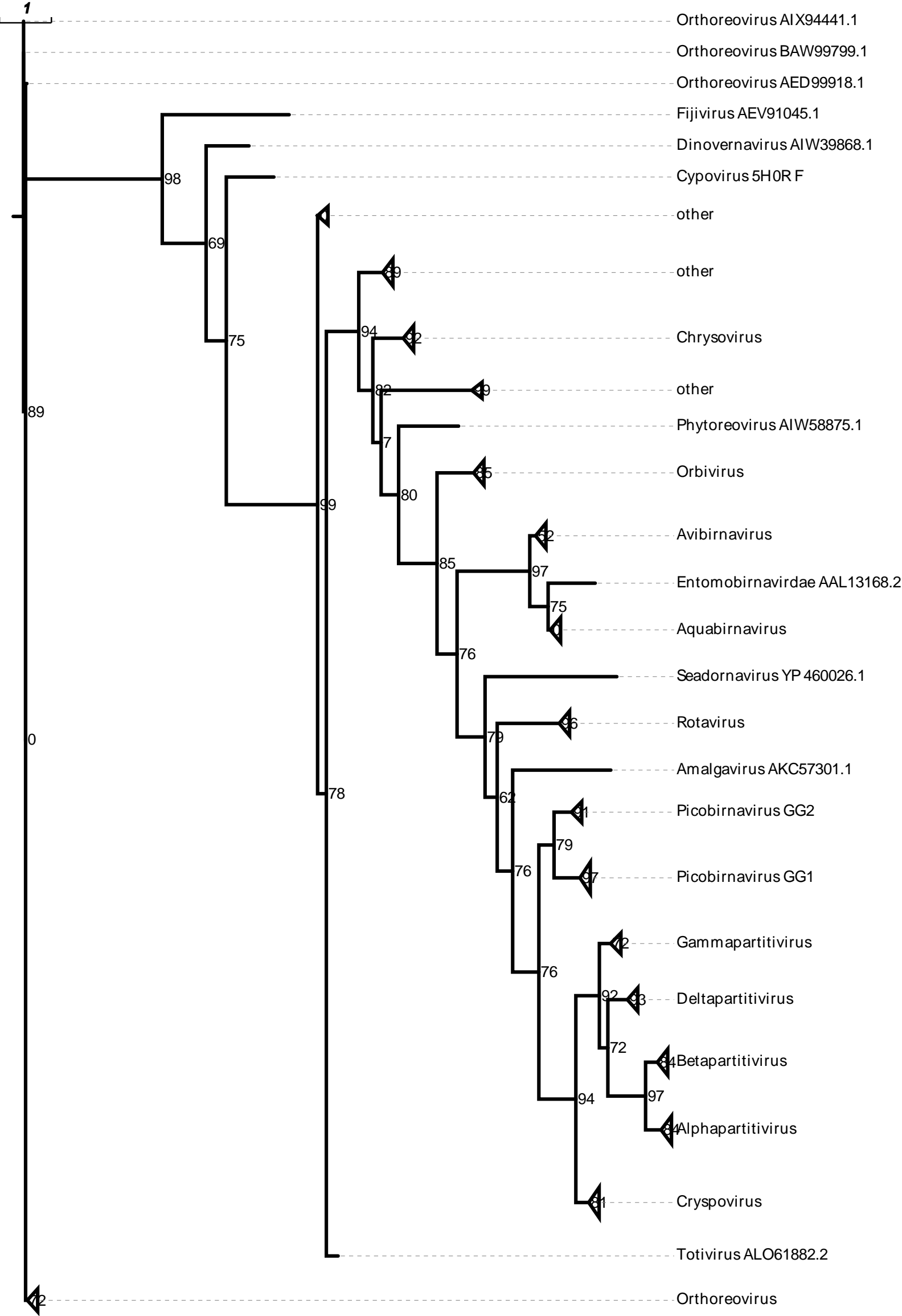

Supplement: Supplementary file 1 [file viruses-10-00685-s001.zip › SI/Figure S3_C.pdf]
