# Supplementary material for: The Challenges of Analysing Highly Diverse Picobirnavirus Sequence Data
Source: Viruses. 2018 Dec 3;10(12):685. doi: 10.3390/v10120685 (PMC6316005; doi:10.3390/v10120685)
Supplement: Supplementary file 1 [file Figure S3_A.pdf › SI/Figure S3_A.pdf]

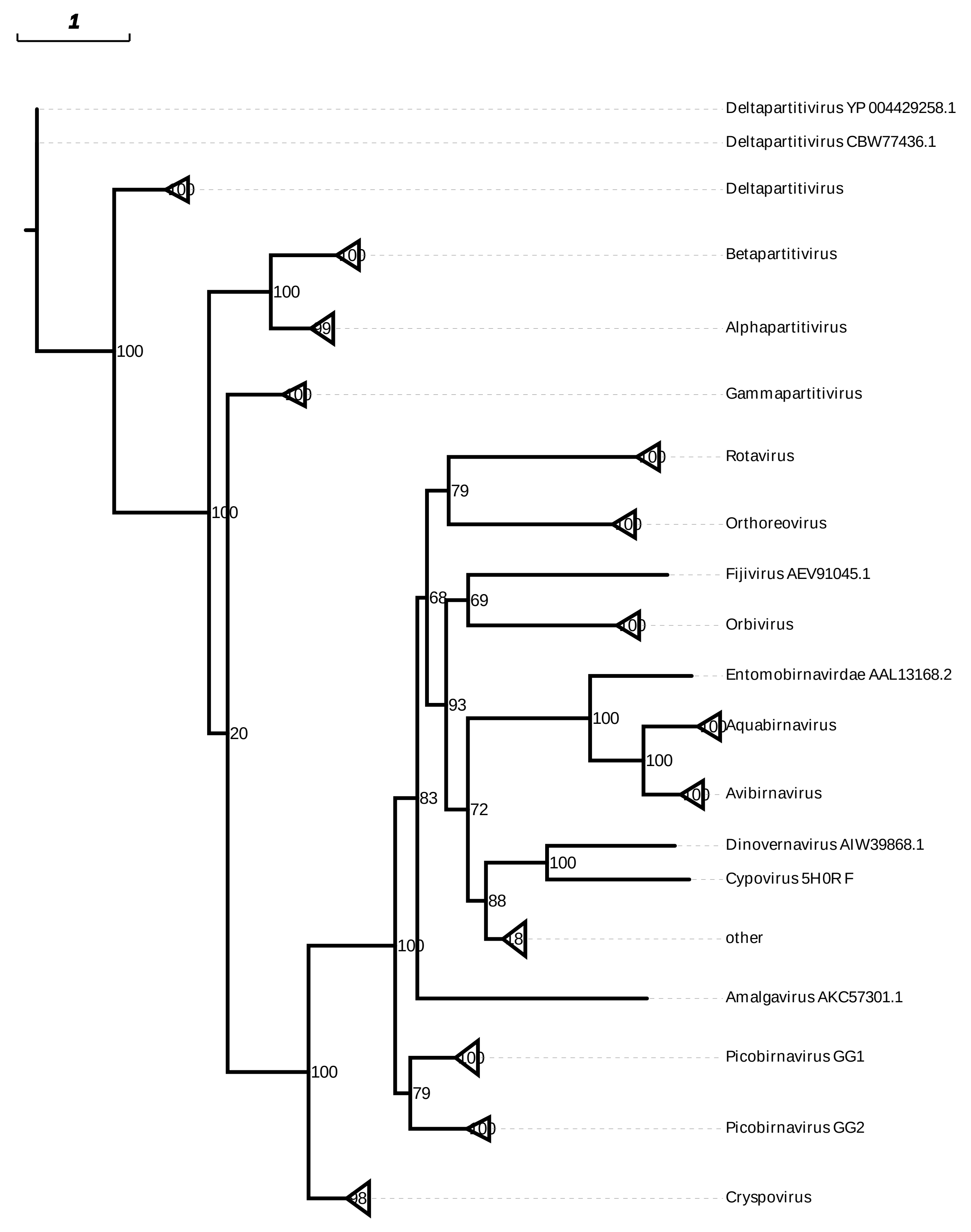
1
Deltapartitivirus YP 004429258.1
Deltapartitivirus CBW77436.1
Deltapartitivirus
Betapartitivirus
Alphapartitivirus
Gammapartitivirus
Rotavirus
Orthoreovirus
Fijivirus AEV91045.1
Orbivirus
Entomobirnavirdae AAL13168.2
Aquabirnavirus
Avibirnavirus
Dinovernavirus AIW39868.1
Cypovirus 5H0R F
other
Amalgavirus AKC57301.1
Picobirnavirus GG1
Picobirnavirus GG2
Cryspovirus
